# Detection of genome-wide methylation changes in bladder cancer by long-read sequencing of urinary DNA

**DOI:** 10.1186/s13148-025-01946-5

**Published:** 2025-08-11

**Authors:** Anshita Goel, Benjamin J. Tura, Joanne D. Stockton, Nicholas Tovey, Luke Ames, Andrew D. Beggs, Maurice P. Zeegers, Nicholas D. James, K. K. Cheng, Richard T. Bryan, Douglas G. Ward, Roland Arnold

**Affiliations:** 1 The Bladder Cancer Research Centre, Department of Cancer and Genomic Sciences, College of Medicine and Health, University of Birmingham, Birmingham, B15 2TT UK; 2 Genomics Birmingham, College of Medicine and Health, University of Birmingham, Birmingham, B15 2TT UK; 3 Department of Epidemiology, School of Nutrition and Translational Research in Metabolism, University of Maastricht, Maastricht, The Netherlands; 4 The Institute of Cancer Research, London, SM2 5NG UK; 5 Department of Applied Health Sciences, College of Medicine and Health, University of Birmingham, Birmingham, B15 2TT UK

**Keywords:** Bladder cancer, Methylation, Long-read sequencing, Nanopore, Urine DNA

## Abstract

**Background:**

Non-invasive urine tests for bladder cancer (BC) could reduce dependence on flexible cystoscopy for diagnosis and surveillance. Most recent developments in urine testing are based on targeted detection of genomic and/or epigenomic markers. We hypothesised that long-read whole-genome sequencing of urinary DNA with direct methylation profiling may allow accurate BC detection and insights into disease biology. However, the feasibility of such an approach has not yet been reported.

**Methods:**

We applied long-read whole-genome sequencing with direct methylation detection to urine cell pellet DNA (ucpDNA) from 21 haematuria clinic patients: 13 BCs and 8 non-BCs. The modkit Hidden Markov Model algorithm was used to define differentially methylated regions across the genome. The ability to discriminate between BC and non-BC, and the cellular pathways affected were tested using PCA, h-clust and GSEA.

**Results:**

We observed global hypomethylation and cancer-specific patterns of promoter hypermethylation in urine from BC patients. Sequencing of a single ucpDNA sample per flow cell yielded read depths of 18-34x; furthermore, BC methylation patterns were also evident with 2–5x multiplex sequencing. Copy number changes were also evident in ucpDNAs from BC patients. A limitation of the study is the small number of samples analysed; however, the detection of cancer-specific events demonstrates the feasibility of the approach, both in single and multiplexed flow-cell runs.

**Conclusions:**

Even at low-read depths, genome-wide methylation patterns in urinary DNA reflect the presence of BC, potentially permitting rapid, non-invasive and cost-effective BC detection.

**Supplementary Information:**

The online version contains supplementary material available at 10.1186/s13148-025-01946-5.

## Background

Patients with symptoms suggestive of BC (e.g. haematuria) are investigated by flexible cystoscopy [1], an invasive, operator-dependent procedure with reported sensitivities and specificities of 81–100% and 64–100%, respectively [2, 3]. Cystoscopy is also used for post-treatment surveillance [1]. Non-invasive, urine-based tests with accuracy reliably equalling or exceeding cystoscopy are a priority to improve early detection and surveillance, while improving patient quality of life and reducing healthcare costs [4].

Numerous studies have highlighted the potential of changes in DNA methylation as urinary biomarkers for diagnosing BC [5, 6]. Since DNA hypermethylation is recognised as one of the earliest events in urothelial carcinogenesis, DNA methylation analysis holds great promise as a tool for the early detection of BC [7]. While commercialised methylation-based BC diagnostic tests show higher sensitivity than urine cytology, they use a small number of methylation markers, e.g. 15 in the case of Bladder EpiCheck [8], and disease complexity may not be captured with targeted methylation panels.

Bisulphite conversion is central to most methylation analyses but leads to DNA damage and loss [9]. Whole-genome sequencing using the Oxford Nanopore Technologies (ONT) platform can detect CpG modifications directly without the need for bisulphite conversion, and at comparable sensitivity and accuracy [10]. The base detection signals can be monitored in real-time during sequencing, and the platform is flexible enough for adoption in a wide variety of clinical/laboratory settings [11, 12].

We hypothesised that genome-wide direct methylation detection by ONT sequencing has the potential to both detect BC and characterise BC molecular biology from ucpDNA, providing differential diagnosis and prognostic and predictive information [13]. To our knowledge, this study presents the first assessment of native methylation calling for non-invasive BC detection. We used ONT data from ucpDNA from 13 patients with non-muscle invasive bladder cancer (NMIBC) (with variable tumour DNA fractions, as determined by GALEAS™ Bladder [14, 15]) and 8 non-cancer patients to define differentially methylated regions (DMRs); the DMRs discriminated cases from controls, showed enrichment of cancer pathways and verified known methylation markers.

## Methods

### Patients and sequencing

Urine samples from 21 patients were collected from haematuria clinics (ethics reference 15/NW/0079) or treatment-naive newly diagnosed BC patients (BCPP, ethics reference 06/MRE04/65) as previously described. The cohort comprised 13 BC cases (all NMIBC, grades 1–3 and stages pTa or pT1) and 8 non-BC patients (6 with no abnormality detected, 1 with kidney stones, 1 with bladder neck stenosis); further details are available in **Supplementary Table S1**. All patients gave written informed consent for urine collection for biomarker research. Details of DNA extraction, sample selection and library preparation are in Supplementary Methods. Libraries were sequenced for 72-h on R10.4.1 PromethION flow cells. All samples had previously undergone targeted sequencing for mutation detection using the GALEAS Bladder panel [15].

### Long-read whole-genome data analysis

Minimap2 [16] was used to align raw reads with base modification tags to the reference human genome (GRCh38), and different modules of samtools (v 1.17) [17] were employed for bam file processing and methylation signal extraction. Modkit (v 0.4.1) was used to quantify 5-methylcytosine (5mC) calls, and subsequently to identify differentially methylated regions (DMRs) by comparing 8 non-BC samples and 13 BC samples using the modkit Hidden Markov Model (HMM). The two-state HMM detects spatially correlated epigenetic changes across the genome. Each genomic position is assigned to one of two hidden states where the two hidden states, "Different" and "Same", indicate that the position is either differentially methylated or not. The DMRs were subsequently filtered as described in Supplementary Methods and methylation scores were extracted using deepTools [18] (v 3.5.2). Mann–Whitney tests were used to select the top 10,479 DMRs (p < 0.05). In order to check that the analysis uncovers disease-specific signals, we performed 50 randomisations of the sample set, each time assigning the samples into groups of 8 and 13 and repeating the whole DMR identification pipeline. GSEA was performed using the MSigDb Hallmark pathways, for the genes with differentially methylated regions in their promoter and/or gene body. To check that the pathways observed were not chance occurrences, 50 sets of 10,479 segments were randomly sampled from the modkit DMR filtered set and GSEA performed for each set. CNVs were called using the QDNASeq package [19] (v 1.40.0) in R (v 4.4.1), and genome-wide copy number burden was calculated as a percentage of the genome. Further details are provided in Supplementary Methods.

## Results

### Generation of long-read whole-genomes from urinary DNA

In total, 21 urine DNAs were whole-genome sequenced with native methylation calling using the ONT platform. Initially, 9 BCs and 5 non-BCs were sequenced at one sample per flow cell, providing median genome coverages of 18-34x. These data suggested that lower read depths might be sufficient for BC detection and a further 8 samples (4 BCs and 4 non-BCs, including a non-BC repeat sample) were multiplexed at 4 samples per flow cell, providing median genome coverages of 2-5x. The mean read length was 1.8 kb (range 0.7 kb to 3.9 kb) **(Supplementary Table S1).** A non-BC sample was sequenced twice (at both high and low coverage) to investigate the effect of read depth on CpG modification fraction and copy number events. The Pearson correlation coefficient between methylation fraction across 42.9 M CpG positions sequenced in both the high and low coverage for this sample was 0.558 (p-value < 2.2 × 10^–16^) **(Supplementary Fig. S1**). When considering the 10,479 DMRs, the correlation coefficient increased to 0.717. Correlation coefficients were also > 0.6 with all non-BC samples and negative correlations were seen with all high VAF BC samples.

### Differential urine DNA methylation in BC and non-BC patients

We identified 0.73 million genomic segments with differential CpG methylation between the BC and non-cancer patients. Further filtering on DMR length, HMM score, modified CpG positions count and modification fraction resulted in 0.2 million segments. Mann–Whitney tests were used to identify a high-confidence set of 10,479 DMRs of which 4048 were hypermethylated and 6431 hypomethylated in BC. To confirm our DMR search strategy, we plotted separately the hyper- and hypo-DMR segments with their respective genomic flanking regions (± 500 bp), observing the expected hyper- or hypomethylated profiles **(Supplementary Fig. S2A-B).** Differential methylation has been reported to appear frequently at transcription start sites, reflecting differential regulation of gene expression between normal and cancer cells [20]; indeed, this was captured by our DMR approach **(Supplementary Fig. S2C).**

We used Mann–Whitney tests to triage DMRs; however, these did not retain statistical significance after Benjamini–Hochberg multiple testing correction, most likely due to the modest sample size, extremely high number of possible DMRs, disease molecular heterogeneity, different stages and grades of disease included, and the variable tumour DNA fraction in patient samples. In order to determine if our observations as a whole do indeed capture biologically relevant signals, we compared the number of detected DMRs to a random background model (50-fold sample randomisation), as well as the number of enriched pathways within these sets (50-fold DMR random selection). The median number of DMRs from these random group comparisons was 140, strongly suggesting that the majority of our 10,479 DMRs are not chance findings **(Supplementary Fig. S3A)**. The median number of significant pathways observed was 0 for the random segment sets, compared to 8 in the actual analysis **(Supplementary Fig. S3B).**

### Global hypomethylation and promoter hypermethylation in BC

The majority of the DMR segments were hypomethylated (61%) in BC, covering 14 Mb of the genome, whereas hypermethylated segments (39%) covered 5 Mb. The average size of hypermethylated segments was 1.2 kb versus 2.2 kb for hypomethylated segments. Hypermethylated segments showed greater absolute changes in methylation levels than hypomethylated segments: average delta values of 2 vs. 0.7, respectively (Mann–Whitney p-value < 2.2e-16).

**Fig. 1:**
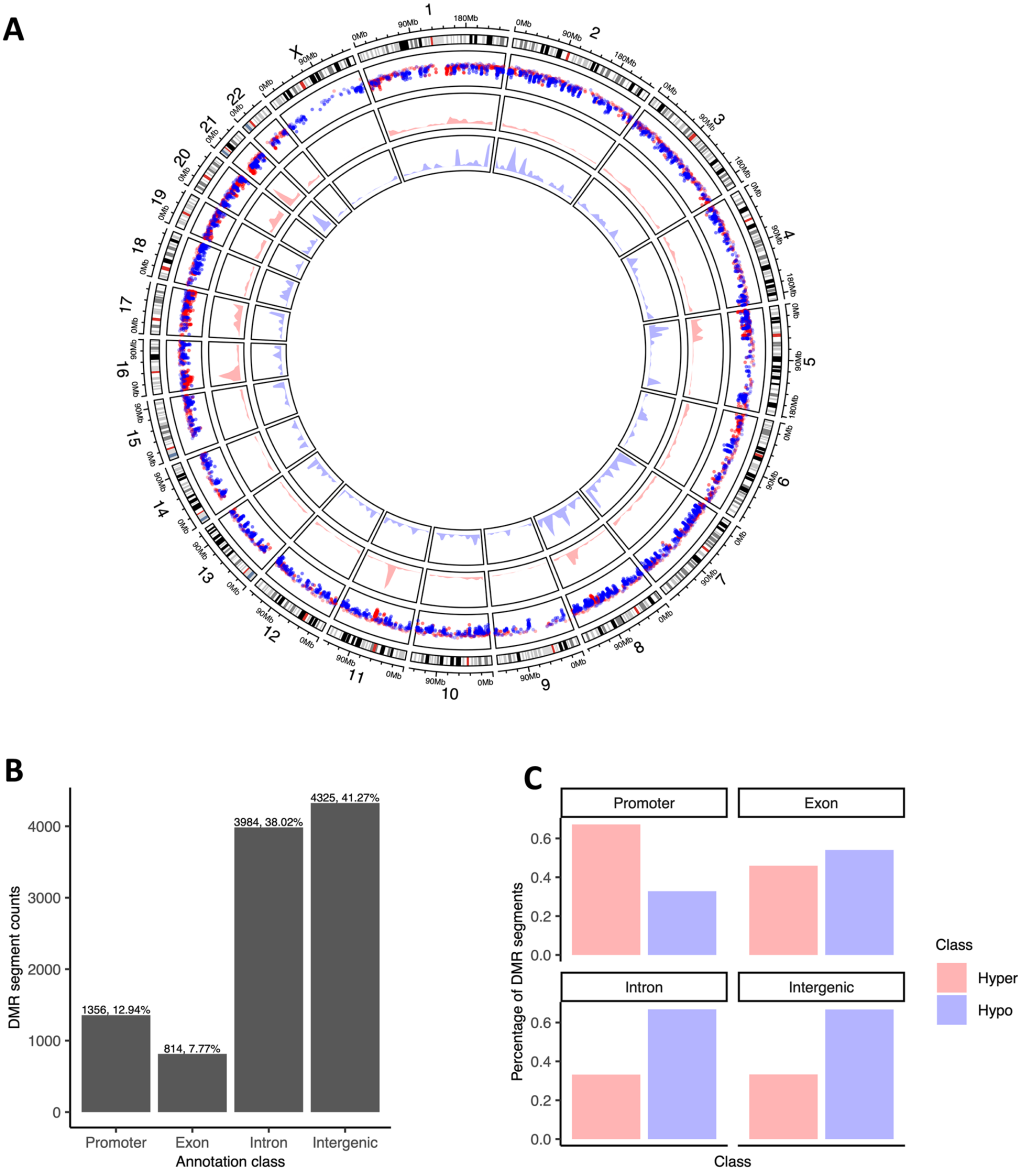
**A** Circos plot of genome-wide methylation and copy number changes in BC patient urinary DNA. The outermost track is the chromosome ideogram. Moving from the outer to the circle centre, the 2nd track is a rainfall plot of DMRs across the chromosomes (red and blue indicate hyper- and hypomethylation, respectively). The 3rd and 4th tracks are the density plots for hyper- and hypomethylated DMRs, respectively. **B** Distribution of DMR counts across the genomic context classes. **C** Comparative distribution of hypermethylated versus hypomethylated segments per genomic context annotation class

DMRs were observed across all chromosomes (Fig. 1A), but their distribution was not directly related to chromosome size or gene content, indicating non-randomness of the methylation events: the chromosomes with the most hypermethylated DMRs were chr1, chr17, chr5 and chr6 (10, 9, 6.5 and 6.5% of all hypermethylated DMRs, respectively), and the chromosomes with the most hypomethylated DMR content were chr2, chr7 and chr8 (9.8, 9.6 and 9%, respectively). Annotation of DMRs for genomic context revealed that 13% were in promoter regions (± 3 kb of TSS for 1036 unique genes), 8% exonic (for 589 unique genes), 38% intronic (for 1677 unique genes) and 41% intergenic (covering 7.9 Mb of intergenic space) (Fig. 1B**)**. DMRs were frequently hypermethylated in gene promoters, whereas in other regions hypomethylation dominated **(**Fig. 1C**)**; hypermethylated DMRs (versus hypomethylated DMRs) were around 4 times more likely to be in promoter regions, whereas hypomethylated DMR segments were 2 × more likely to be in intergenic regions **(Chi-square test p-value < 2.2e-16; Supplementary Fig. S4).**

### DMRs in urine DNA distinguish bladder cancer patients from non-cancer patients

**Fig. 2:**
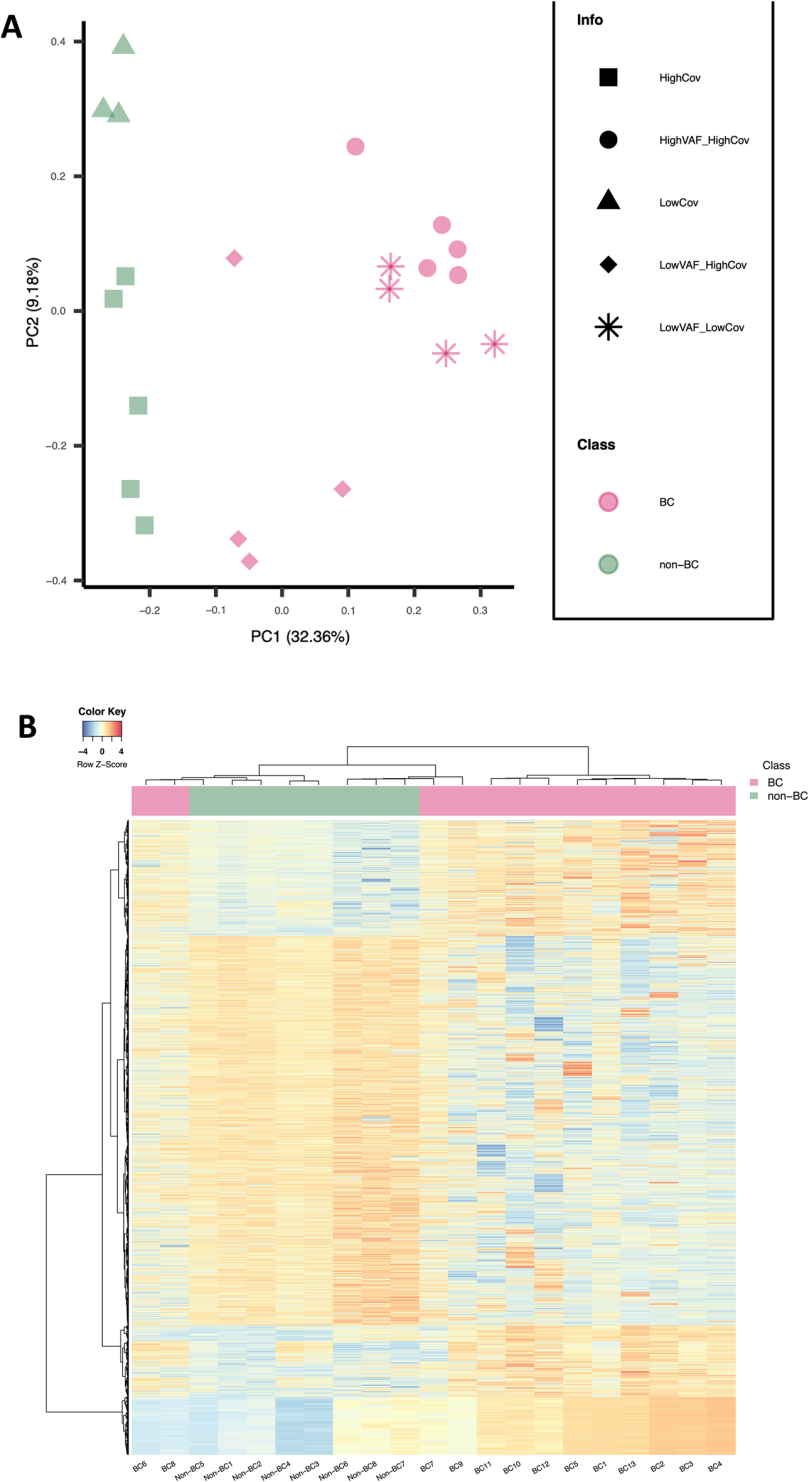
Principal component analysis (PCA) and hierarchical clustering of BC and non-BC urine DNA methylation. **A** PCA plot with the X- and Y-axes representing PC1 and PC2, respectively. Non-BCs are represented by green and BCs by pink symbols. The shapes of the data points indicate whether the samples have low or high sequencing depth and indicate low or high variant allele frequency cases for BC samples. **B** Heatmap showing hierarchical clustering of DMRs. X- and Y-axes represent the samples and DMR segments, respectively

**Fig. 3:**
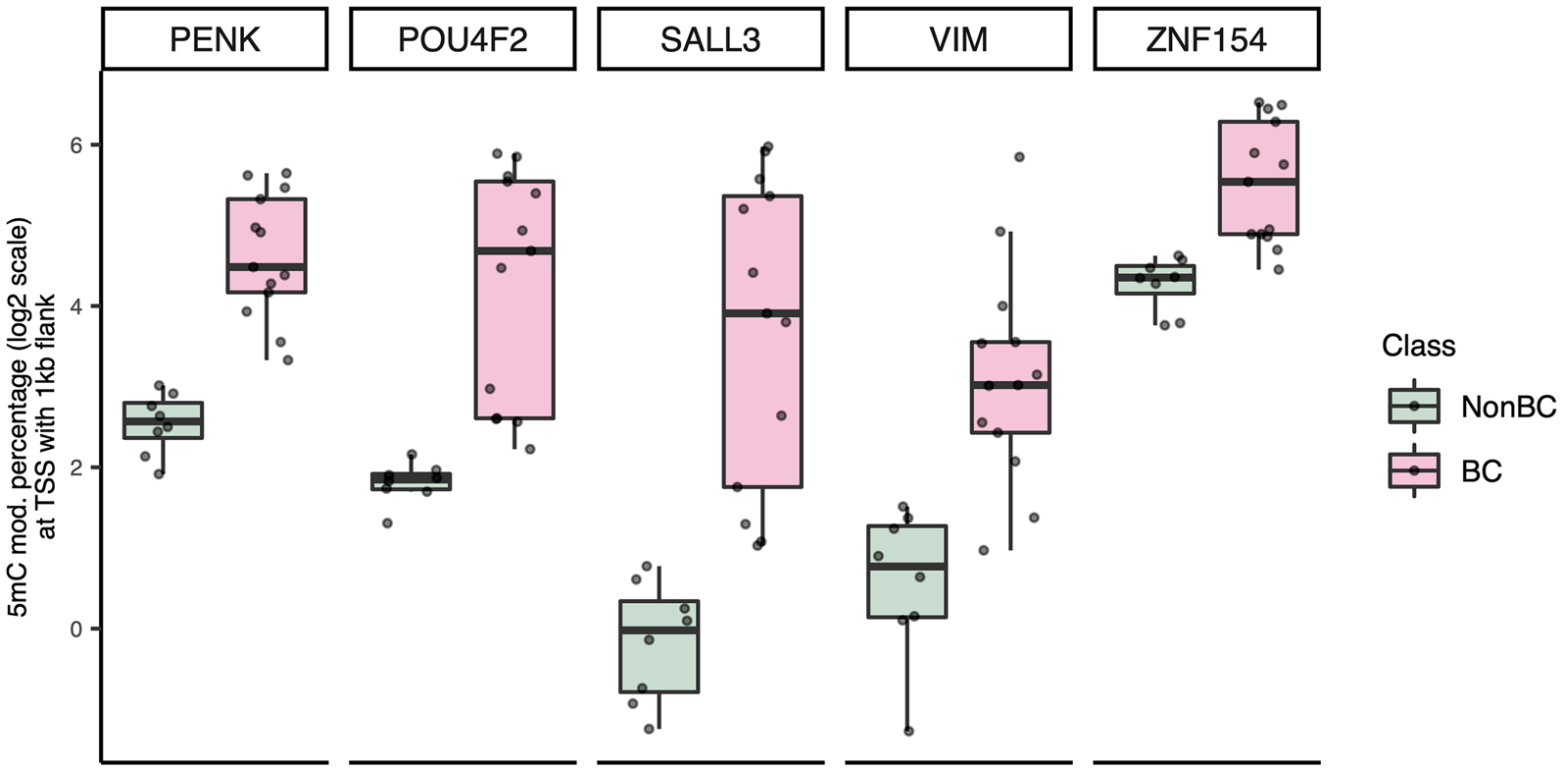
Nanopore methylation data across known BC biomarkers. The boxplot shows the methylation status (determined by ONT direct calling) for 5 gene promoters reported as hypermethylated in BC in a recent systematic review. The 5mC modification fraction (aggregate signal value for all constituent CpG positions in log2 scale; Y-axis) in the 1 kb upstream and downstream of TSS (transcription start site) was plotted separately for non-BC and BC samples, for the five different biomarkers (X-axis). The dots represent the non-BC (n = 8) or BC (n = 13) samples in the cohort

Principal component analysis of the DMRs separated BC and non-cancer urine samples **(**Fig. 2A**).** Five BC samples with high-coverage and high tumour content (VAFs > 30%) clearly separate from the high-coverage non-BC samples in PC1. Four NMIBC samples with low coverage and VAFs of 13–25% segregate with the high VAF high-coverage NMIBC samples, demonstrating that DMR segments can provide cancer-specific signals even in samples with low sequencing depth. Similarly, the low-coverage non-cancer samples segregate with the high-coverage non-cancer samples. Four high-coverage NMIBC samples with lower VAFs lie between the non-BC and higher VAF samples. We also analysed the data by h-clust obtaining a BC cluster and a predominantly non-BC cluster (Fig. 2B). The BCs which cluster with the non-BCs are the same low VAF high-coverage BCs which are close to the non-BCs in the PCA. Commonly reported hypermethylation markers [21] behave as expected in our data **(**Fig. 3**).**

All BC urine samples were positive for ≥ 1 SNV when previously deep-sequenced with GALEAS Bladder. Although the ONT sequencing read depth is insufficient for sensitive SNV detection, 21 of the 31 SNVs detected in the BC urines by GALEAS Bladder (68%) were also supported by ≥ 1 read in the 9 high-coverage BC urine samples, and 12 out of 29 (41%) in the low-coverage BC urine samples.

### Gene set enrichment analysis (GSEA) of DMRs

**Fig. 4:**
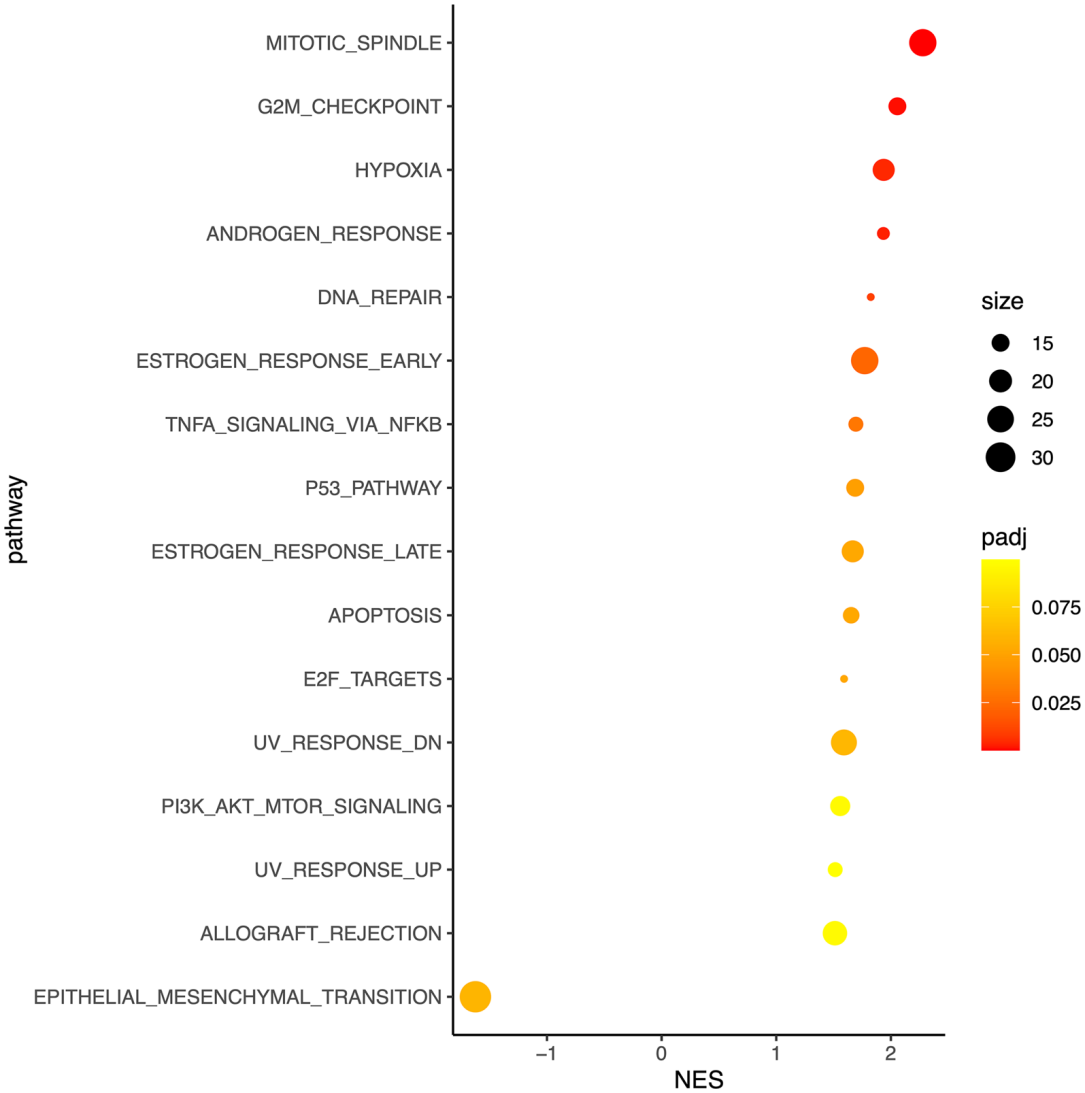
Gene set enrichment analysis (GSEA) of DMRs using MSigDb Hallmark pathways. Of the 50 pathway sets, 8 were found to be significant at threshold of adjusted p-value < 0.05 and normalised enrichment score (NES) >  = 1.5 or <  = − 1.5. The above plot shows all the pathways crossing the NES threshold irrespective of adjusted p-value. The X- and Y-axes denote NES and the pathway names, respectively. The size of the given dot for a given pathway is indicative of the number of genes (legend “size”) observed from the pathway, and the colour is indicative of the adjusted p-value (legend “padj”)

The DMR segments included the promoter and/or gene body regions of 2,769 genes—we ranked these genes by methylation delta value to reflect the difference between BC and non-BC methylation. GSEA using the ranked methylation data on the 50 Hallmark (MSigDb) pathways revealed positive enrichment of 8 pathways (adjusted p-value < 0.05) for hypermethylation signal **(**Fig. 4** and Supplementary Table S2).** The most significant pathways were mitotic spindle, G2M checkpoint and hypoxia. These data suggest that genome-wide urine DNA methylation patterns could be used for the non-invasive molecular characterisation of primary bladder tumours**.**

### Long-read sequencing provides comprehensive coverage of methylation in repetitive elements

In each sample, we observed a uniform read depth regardless of the repetitive sequence content (average difference between repetitive and non-repetitive: 0.028x, with standard deviation of 0.036), demonstrating that long-read sequencing enables unbiased investigation of these regions with reliable coverage after alignment. This ability to circumvent potential blind spots caused by repetitive elements—an issue potentially encountered in short-read sequencing due to mapping difficulties [11]—proved advantageous in our analysis. Notably, approximately 59% of the sequence within intergenic region DMRs, which tend to be hypomethylated in BC, consisted of repetitive elements, compared to 27% in exonic DMRs and 30% in promoter DMRs. **(Supplementary Fig. 5A)**. Furthermore, the repetitive element content of hypomethylated DMRs was significantly higher than the hypermethylated DMRs (Mann–Whitney p-value < 2.2e-16) **(Supplementary Fig. 5B)** and the difference in distribution of the repetitive element content in the two DMR classes was more prominent in promoter and exonic regions **(Supplementary Fig. 5C).**

### Copy number variants in BC patient ucpDNA

**Fig. 5:**
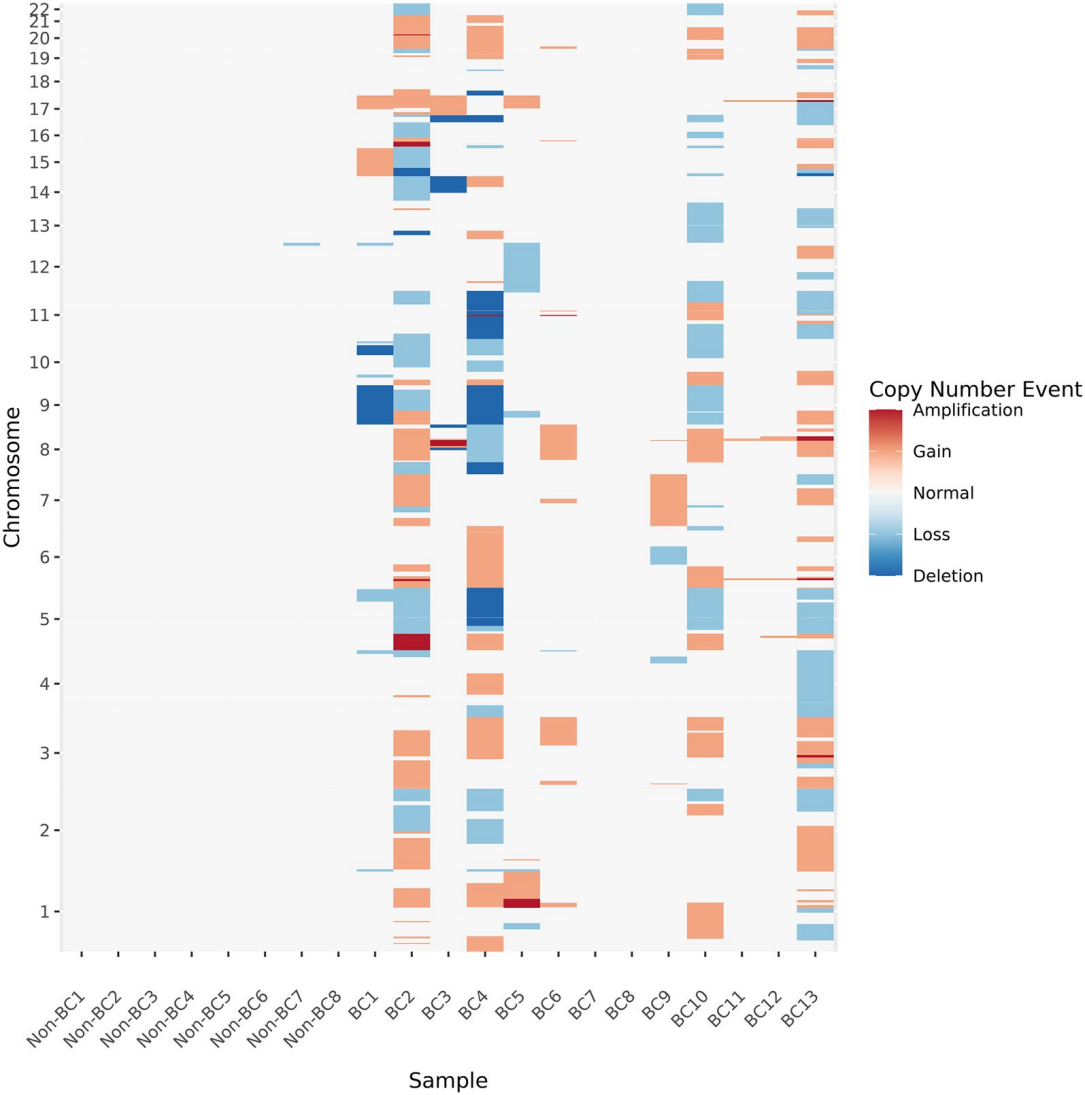
Copy number changes in DNA from the urine of BC patients and control subjects. The figure shows copy number event calls made in non-bladder cancer (non-BC) and bladder cancer (BC) samples. The Y-axis has the chromosome locations (chr1-22 in the positive direction), with samples arrayed along the X-axis

Since ucpDNA is extracted from an admixture of healthy and tumour cells, we hypothesised that copy number detection methods can be effectively applied, provided the tumour content is not too low. High VAF BC urine samples were easily distinguishable from non-cancer patients in genome-wide karyotype profile plots, but less so for low VAF cases **(**Fig. 5** and Supplementary Fig. 6A-D);** the BC urine genomes harboured more CNVs than the non-BCs (20.8% vs. 0.04% of the genome altered; Mann–Whitney p-value 0.001) **(Supplementary Fig. 6E**). Amplification of chr8q was observed in 61% of BCs, and across these samples, the chr8q22.2 region was consistently altered. Chr9p aneuploidy was observed in 46% of samples. On chr5, the p arm tended to show gains (38%), while the q arm tended to be lost (38%). Chr17 showed a similar pattern with losses frequently seen on the p arm (38%) and typically gains on the q arm (Fig. 1A). The changes on chr9, chr5 and chr17 were not detected in any low VAF BC samples.

## Discussion

We present a combination of ONT whole-genome sequencing and a computational strategy to identify and characterise methylation regions that discriminate between urinary DNA from patients with and without BC. These approaches revealed discriminative signals over a range of tumour fractions and sequencing depths. These data also indicate that promotor methylation patterns in urinary DNA can reveal biological processes in the associated primary tumours. The CNV and methylation changes reported here are consistent with those in the literature, and it is likely that (at least in urine DNAs with high tumour fraction) tumour subtypes, therapeutic targets and biomarkers for urine-informed plasma ctDNA monitoring can also be identified [22]. An advantage of using ONT whole-genome sequencing for methylation profiling (versus standard next generation sequencing) is that it can comprehensively profile all CpG islands, including those harboured within large structural variants or polymorphic repetitive elements that are hard to resolve using short reads [23]. Furthermore, whole DMRs can be sequenced end-to-end in a single read with ONT sequencing, rather than being inferred from multiple short reads as in standard next generation sequencing. Thus, this is a single molecule technique with the potential to identify coordinated and complex methylation changes in DNA from a single cancer cell—the proverbial “needle in the haystack” of liquid biopsy analysis [24].

This is a proof-of-principle study which, to our knowledge, is the first to describe the use of ONT long-read sequencing of urine cell pellet DNA methylation for the detection of BC [25]. Theoretically, this approach would also work with urine or plasma cfDNA [26], although for this study we chose ucpDNA due to the high DNA yields and longer read lengths achievable and, despite contradictory reports, there may be little difference between urine ucpDNA and ucfDNA for urothelial cancer detection [27]. The data presented here show that long-read sequencing of urine DNA is highly promising for obtaining comprehensive genomic and epigenomic information about BCs. Such information may extend beyond that gained from smaller biomarker panels, potentially improving accuracy in the detection setting. Although already under way [13], further work is required to establish the utility of methylation profiles for BC subtyping, and larger cohorts need to be analysed so that class prediction models can fully exploit the complexity of genome-wide DNA and epigenomic signatures. While our current sample size is too small to generate a supervised class prediction model to detect BC that covers sufficient patient heterogeneity, the observed discrimination clearly points towards feasibility of such a model with a larger training-set at hand. Furthermore, a better differential scoring scheme to identify individual DMRs needs to be developed in the future; while our step-wise selection process did identify biologically plausible differences between cancer and normal, individual DMRs might still be spurious—which can be addressed when larger data-sets become available.

## Conclusions

Native methylation analysis of urine DNA by long-read sequencing is a viable approach for non-invasive BC detection. As low-read depths (which could be obtained in a few hours [28]) appear sufficient, this approach is potentially fast and uses portable inexpensive instrumentation suitable for near-patient testing. The resulting data contain a wealth of information which, in combination with class prediction modelling, may yield highly accurate diagnostic and prognostic information.

## Supplementary Information

Additional file 1.

Additional file 2.

Additional file 3.

## Data Availability

Data are available from the corresponding author upon reasonable request.
