# Supplementary material for: Detection of genome-wide methylation changes in bladder cancer by long-read sequencing of urinary DNA
Source: Clin Epigenetics. 2025 Aug 11;17:141. doi: 10.1186/s13148-025-01946-5 (PMC12337379; doi:10.1186/s13148-025-01946-5)
Supplement: Supplementary file 1 — Additional file 1. [file 13148_2025_1946_MOESM1_ESM.pdf]

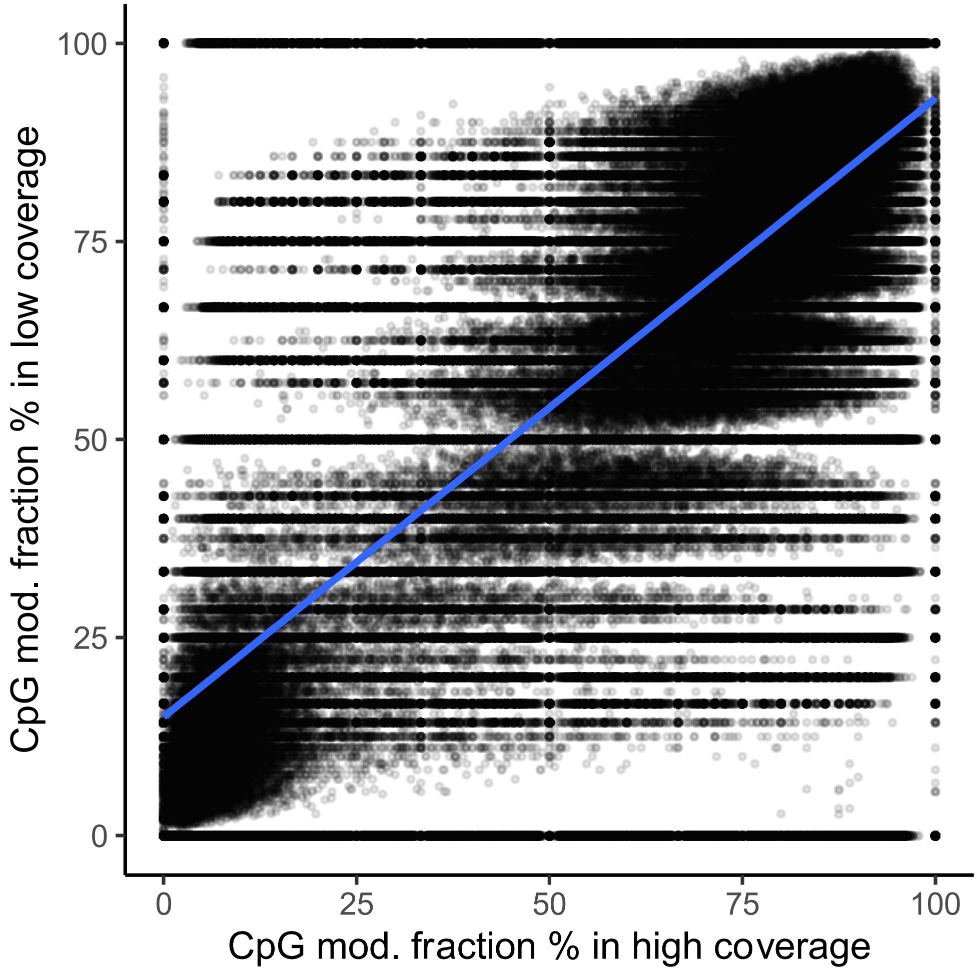

Supplementary Fig S1 : Correlation plot for 5mC methylation fraction across 42.9 million CpG positions sequenced in replicate for the sample Non-BC1. Each point indicates modification percentage for a single CpG position. X- and Y- axes are for the high and low coverage sequencing replicate, respectively. The blue line is the regression line fitted by linear model method. The Pearson correlation coefficient between the high and low coverage non-BC1 sample was 0.558 (p-value <2.2x10-16).

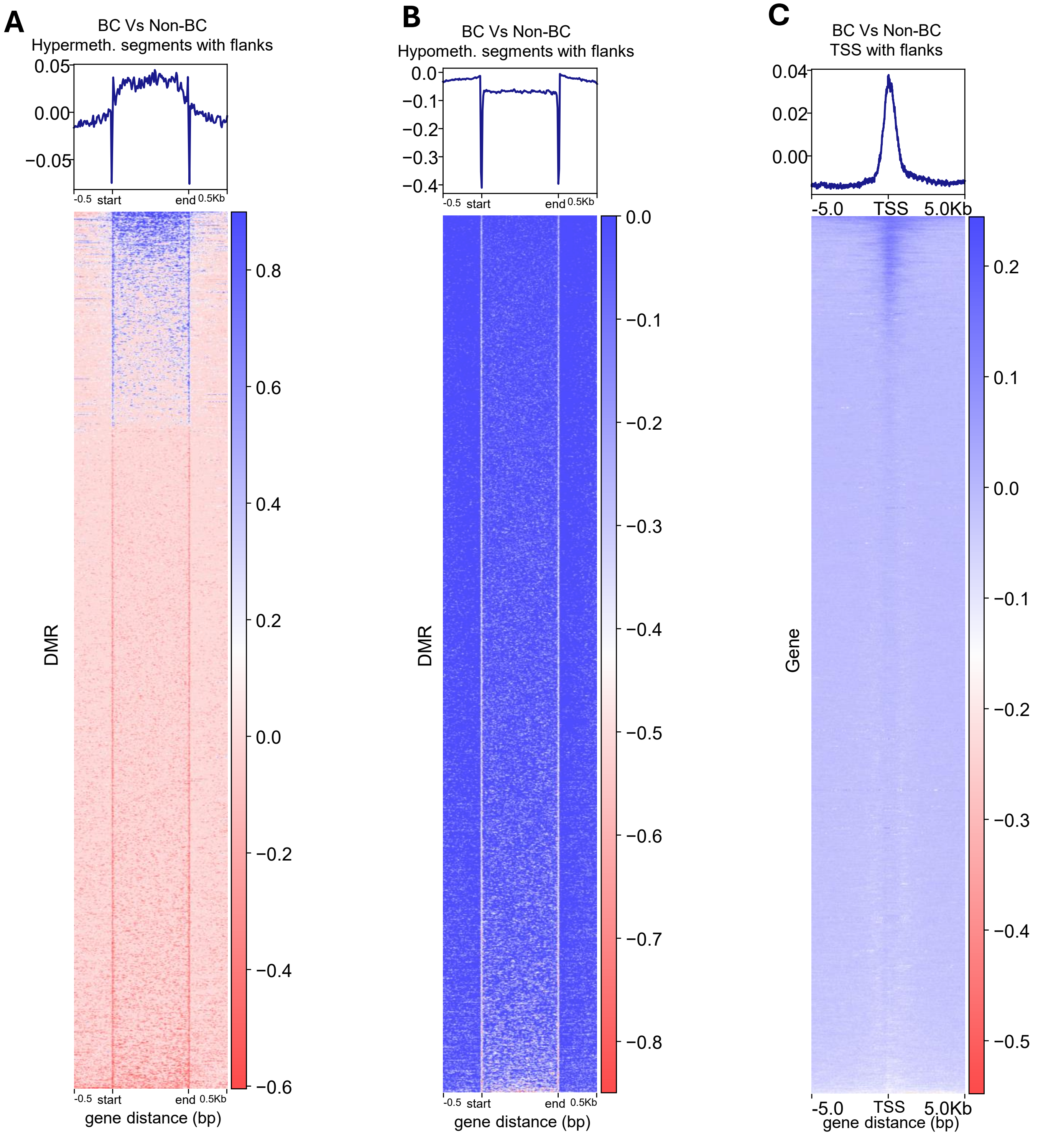

Supplementary Fig S2 : Genome-wide profile of DMR segments in BC Vs Non-BC samples. Comparative bigwig signals were calculated for BC samples (n=13), by comparing to non-BC samples (n=8). DMRs were separated into hyper **(A)** or hypo **(B)**, scaled to 1kb fixed length, aligned and the bigwig signal profile plotted with ±500b genomic flanks. **Fig C** shows 5mC levels across transcription start sites with ±5kb flanks across all genes. Y-axes indicate the log2 ratio of BC vs. Non-BC methylation fraction. The heatmaps show average methylation fraction for each DMR or TSS across the 21 samples (blue = hyper in BC, red = hypo in BC).

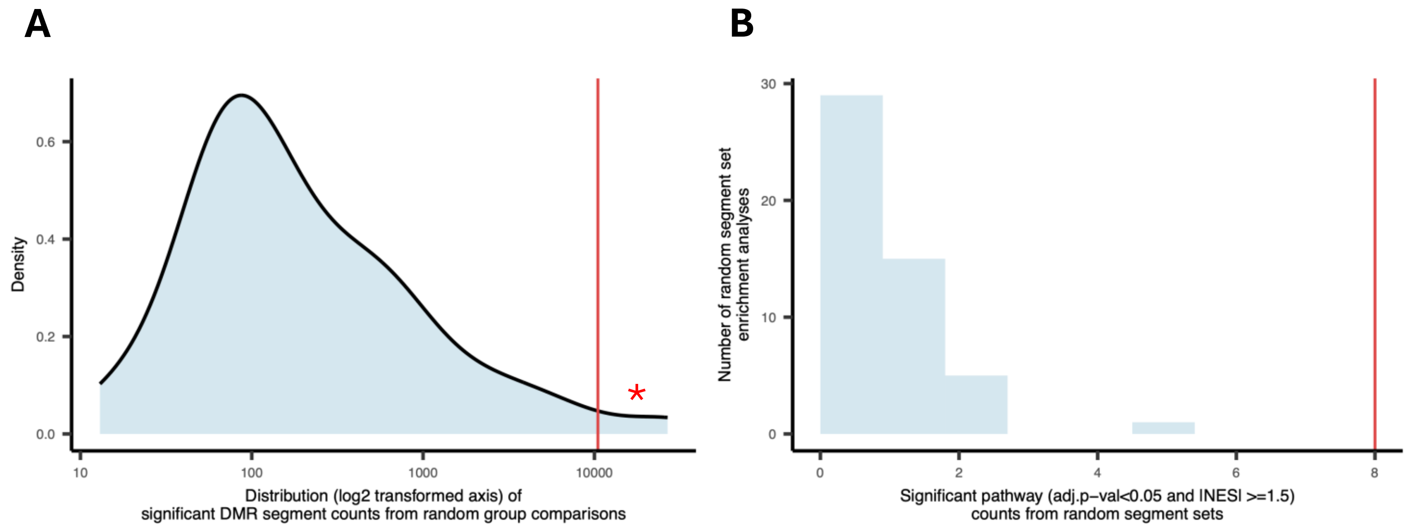

Supplementary Fig S3. Random background models.

A. The distribution of the number of DMRs in 50 random group comparisons (x-axis: number of DMRs detected, y-axis: "Density" = relative likelihood). The red line indicates the number of DMRs in the actual comparison. The red asterisk indicates one randomised sample that scored higher than the non-randomised data. In this randomisation the n=8 group contained 7 BC samples (average VAF = 38%) and the n=13 group contained 7 non-BC and 6 BC samples (average VAF = 13%). **B.** The number of enriched pathways in 50 random selections of 10479 segments. The red line shows the number of pathways from the actual GSEA comparison.

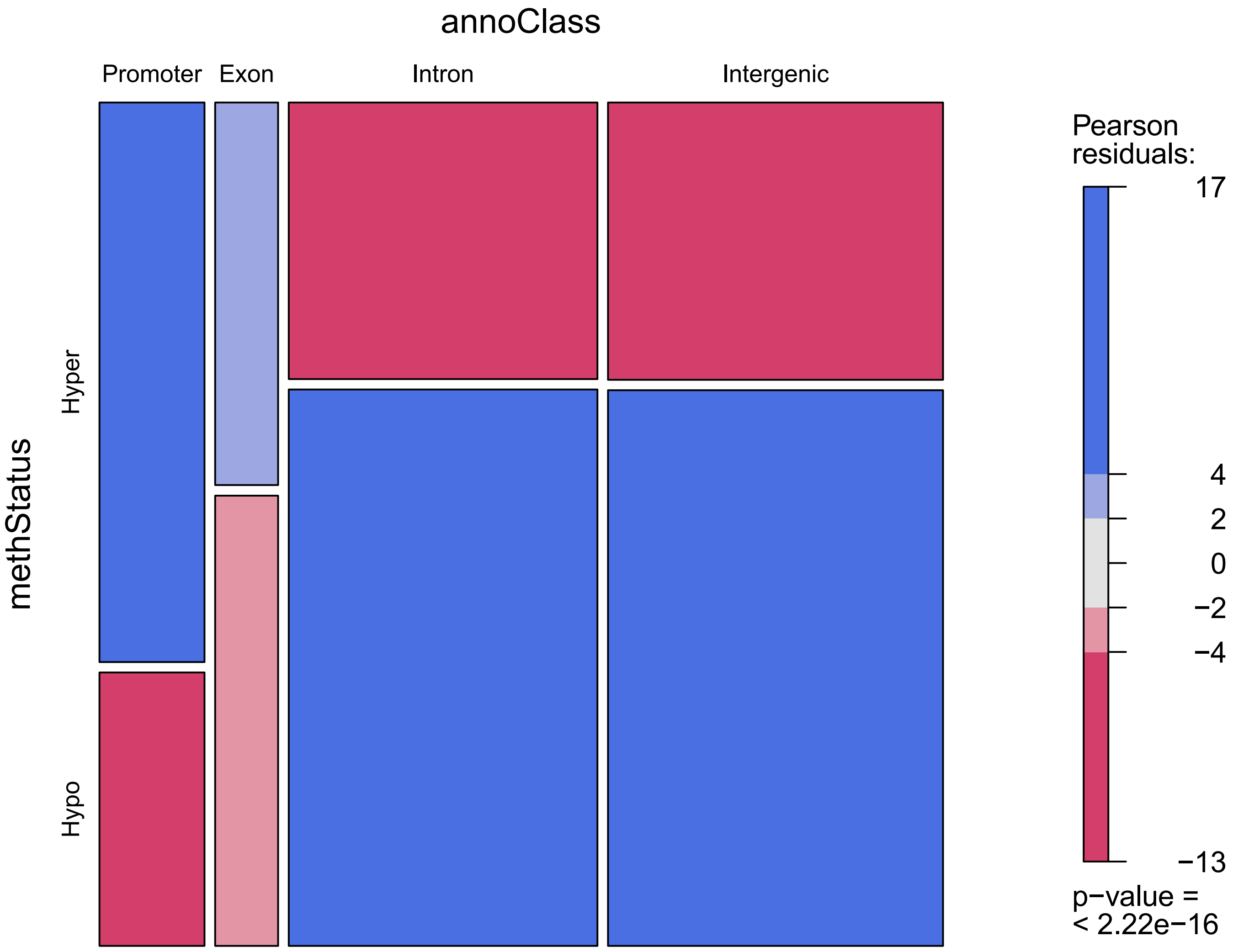

Supplementary Fig S4 : Mosaic plot depicting Pearson residuals from Chi-square comparison of Hyper- versus Hypo- methylated segment counts across the genomic annotation classes

The vertical colour ribbon legend on the right hand-side indicates colour code for Pearson residuals: intense blue indicates more than expected and intense red indicates less than expected. The X-axis of the mosaic plot is for the genomic context annotation classes: “Exon”, “Intergenic”, ”Intron” and “Promoter”. The Y-axis denotes “Hypo” or “Hyper” DMR segments. Only the “Promoter” class has more than expected hyper-methylation. All other genomic context annotation annotation classes have more than expected hypo-methylation.

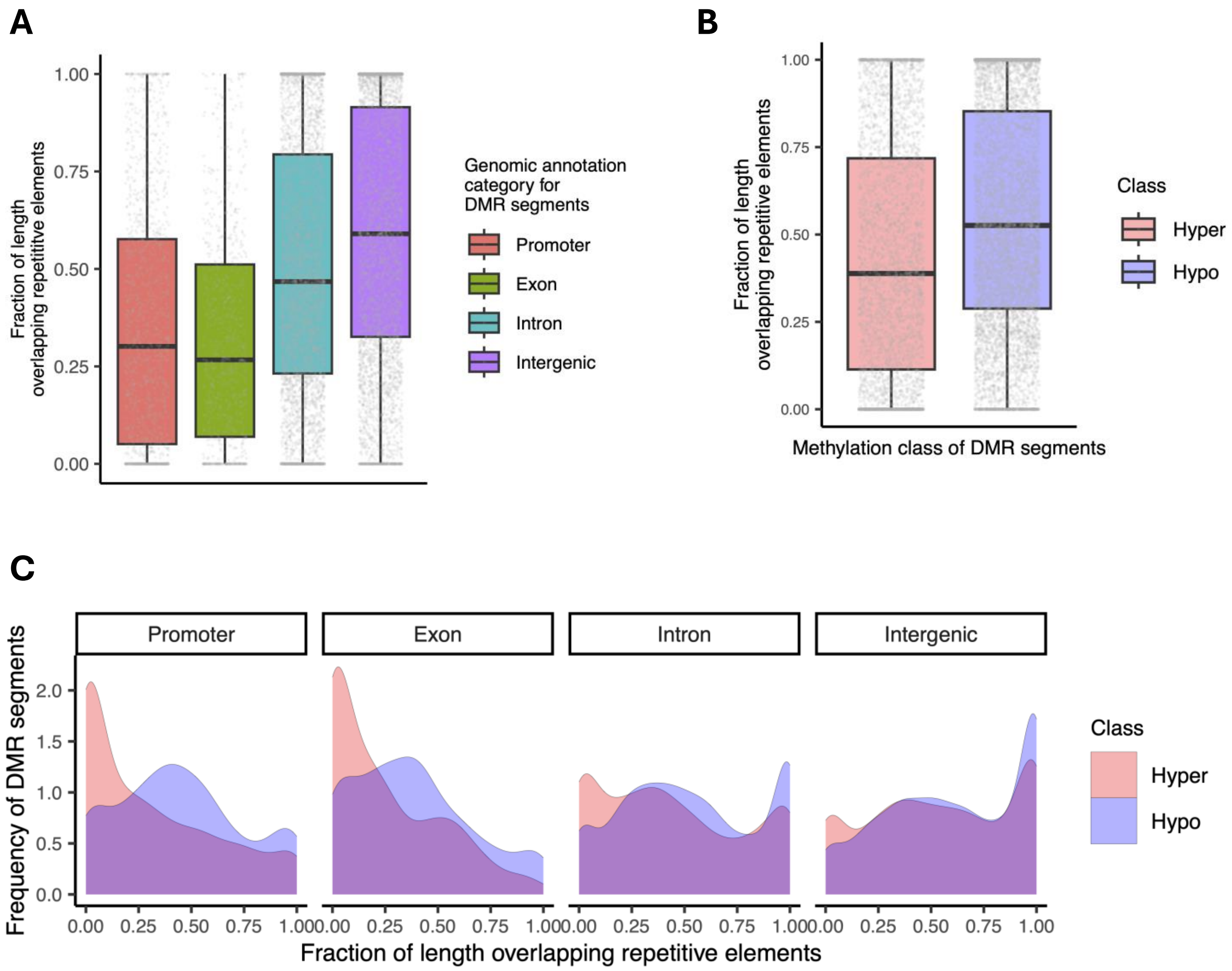

Supplementary Fig S5 : Repetitive element content within DMRs

A. – Boxplot for distribution of DMR length fraction occupied by repetitive elements, per genomic context annotation class. X-axis is the genomic context annotation class and Y-axis is the DMR length fraction overlapping RepeatMasker annotated elements. **B.** – Boxplot here is showing the repetitive element content with respect to hyper- and hypo- methylated DMRs. Y-axis is the DMR length fraction overlapping repetitive elements. Each dot in both **A.** and **B.** represent an individual DMR segment.

C. – Density plots individually for hyper- and hypo- methylated DMR segments across the genomic context annotation classes. The X-axes (value ranging from 0-1) in each sub-plot is the DMR length fraction overlapping repetitive elements. The Y-axis is the density of the DMR segments observed across the range of repetitive element overlap fractions.

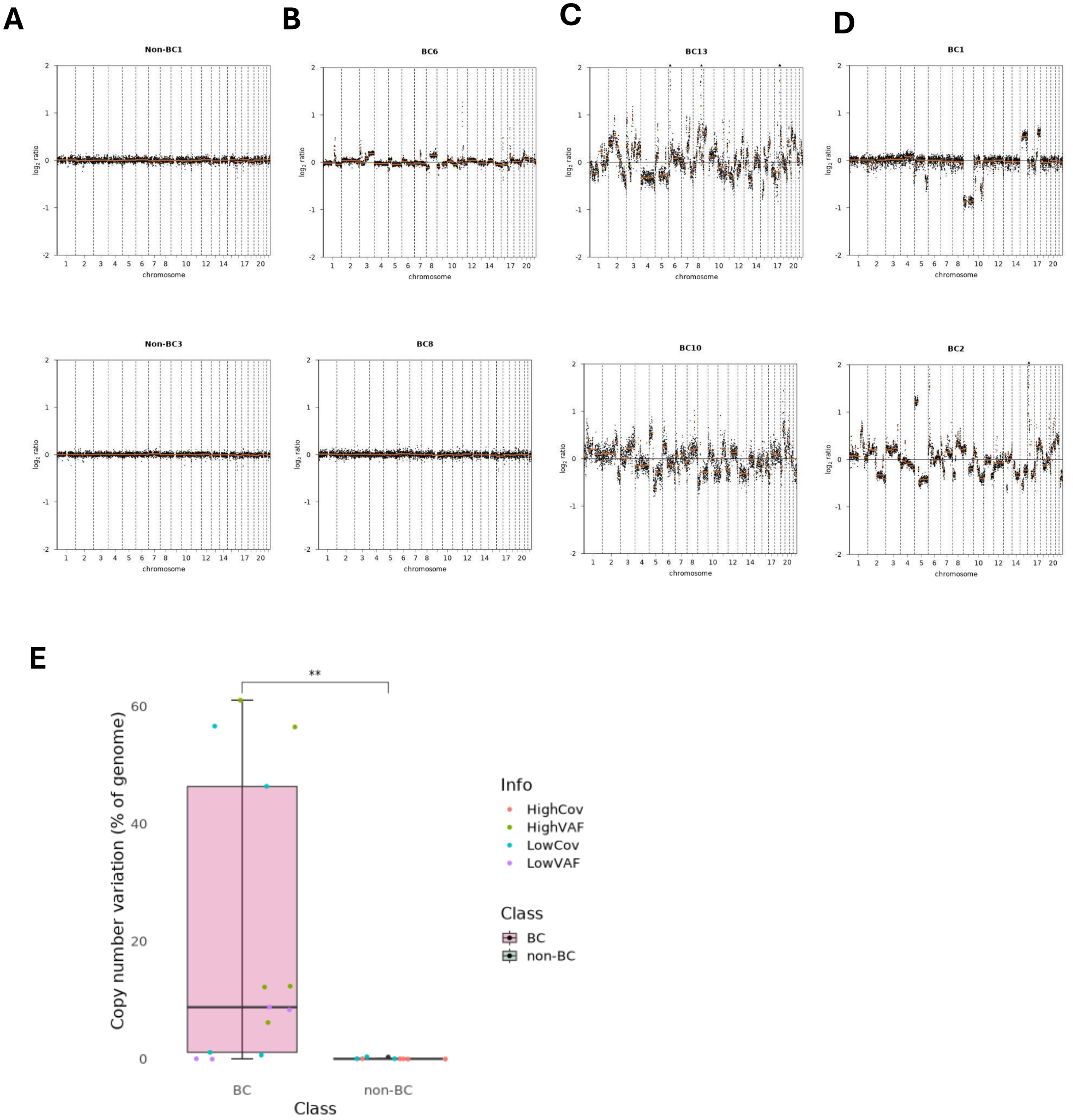

Supplementary Fig S6 : Copy number events observed in long-read whole-genome sequencing of urinary DNA. Two examples are shown of karyotype plots for genome-wide copy number variation from **A:** non-BC, **B:** low VAF BC, **C:** low coverage BC, and **D:** high VAF BC samples. **E**. Copy number events observed in non-BC and BC samples have been transformed to percentage genome altered and represented as a boxplot. The Mann-Whitney p-value for the difference between the non-BC (n=8) and BC (n=13) groups was p = 0.0013.
