# Supplementary material for: Detection of genome-wide methylation changes in bladder cancer by long-read sequencing of urinary DNA
Source: Clin Epigenetics. 2025 Aug 11;17:141. doi: 10.1186/s13148-025-01946-5 (PMC12337379; doi:10.1186/s13148-025-01946-5)
Supplement: Supplementary file 2 — Additional file 2. [file 13148_2025_1946_MOESM2_ESM.docx]

**Supplementary Methods:**

**Sequencing library preparation**

Urine samples (30-50 ml) were centrifuged at 2000 x g for 10 minutes on the day of collection, cell pellets stored at -80°C, and DNA subsequently extracted using the Quick-DNA Urine Kit (Zymo Research #D3061) and quantitated using the high-sensitivity dsDNA Qubit kit (Thermofisher #Q32854). For each sample, 500 ng of DNA was sheared to <10 kb using G-tubes (Covaris #520079) and sequencing adapters ligated using the LSK-114 kit.

**Long-read whole genome sequencing data analysis**

Minimap2 (v 2.24) was used to align raw reads with base modification tags to the reference human genome (GRCh38) and different modules of samtools (v 1.17) were employed for processing the BAM files for the recommended steps of methylation signal extraction. The final BAMs were then used to quantify 5mC calls in bedMethyl format using modkit pileup (v 0.4.1). Modkit DMR (v 0.4.1) was used to identify differential methylation regions (DMRs) by comparing 8 non-BC samples and 13 BC samples. The following filtering criteria were applied to retain high-confidence DMRs: a) ≥ 10 CpGs per segment, b) segment length ≥ 300 bp, c) HMM score > 100, and d) base modification fraction > 0.5 in either BC or non-BC. The remaining DMRs (0.2 million segments) were used to construct a matrix of methylation scores across samples using deepTools (v 3.5.2). To identify the most significant DMR segments the Mann-Whitney test was applied comparing scaled scores between BC and non-BC samples and selecting only those segments with p-value < 0.05. The final set of 10,479 DMRs were used for principal component analysis (PCA) to differentiate between BC and non-BC urine samples. ChIPseeker (v 1.40.0) was used to annotate the DMR coordinates for genomic context.

**Status of known biomarkers in nanopore methylation data**

For the five well-known biomarkers of hypermethylation in bladder cancer, their Transcription Start Site (TSS) were identified using the MANE (Matched Annotation from NCBI and EMBL-EBI) select transcript (GRCh38 genome version, UCSC Table Browser track) and then a genomic coordinate of 2kb span with TSS at the centre was used to extract per CpG position 5mC modification fraction in the Non-BC and BC ONT whole-genome sequencing. 5mC modification fractions were used to generate boxplots for BC and non-BC samples.

**Pathway analysis**

Genes with DMRs detected in either their promoter (3kb flank of TSS) and/ or gene body (exon/ intron) regions were used to perform Gene Set Enrichment analysis (GSEA) using the BC vs. non-BC 5mC modification fraction delta value for ranking. For genes with more than one qualifying DMR segment, the segment with maximum delta value was chosen as representative rank for that gene. In total, 2769 genes were ranked and analysed using fgsea (v 1.30.0) on the MSigDB hallmark pathway set (release 7.5.1) to identify statistical significance at normalised enrichment score (NES) >= 1.5/ <= -1.5, and adjusted p-value <=0.05.

**Repetitive element content assessment within DMRs**

The final set of DMR (10,479) coordinates were used to calculate overlap with the known repetitive element content in the human genome (UCSC RepeatMasker annotation; v October 2022). AnnotationHub (v 3.12.0) was used to retrieve the repetitive elements coordinates, and GenomicRanges (v 1.56.1) was used to calculate the overlap with DMRs. Mann-Whitney tests were used to calculate the significance of repetitive element content (overlap fraction) difference between hyper- and hypo-methylated DMRs.

**Random Background Models**

We used two different randomisation strategies to assess if the overall findings of the DMR analyses truly capture disease specific differences rather than random findings due to the high number of potential DMRs. In one, the sample assignment to the test and control groups was randomised and the DMR calling process repeated. In the second, random segment sets were used to perform gene set enrichment analyses.

Randomised group comparisons for DMR calling - Here the two groups: test (BC) and control (non-BC), retained the size of the original comparison (i.e. 13 and 8 respectively), but the sample assignment to the groups was done by shuffling the total set of 21 samples. 50 such iterations were carried out, and for each comparison modkit DMR run, segment filtering steps and Mann-Whitney based comparison were carried out to count the final number of significant segments obtained.

Random segment set selections for gene set enrichment analyses - Here, the original comparison's filtered set from modkit DMR (~200k segments) was used to randomly select out 10,479 segments (same number as Mann-Whitney in the original comparison), and then these were subjected to gene set enrichment analysis. 50 such iterations were carried out and the number of Hallmark pathways (MSigDb) obtained for each random segment set were counted.

**Copy number analysis**

Copy number was called using QDNASeq (v 1.40.0) within R (v 4.4.1). BAM files were annotated with discrete (500kb-sized) bins. Reads were counted in each bin to obtain estimates of copy number and corrected using a non-parametric, multivariate, local regression function (LOESS) to account for GC content and mappability. A blacklisting procedure based on anomalous regions derived from short-read mapping was used. Although it is probable that the long reads used here should produce better mapping in repetitive regions (centromeres, telomeres, satellites) that tend to be excluded in this step, the blacklisting was maintained since the increase in accuracy for our samples has not yet been proven. QDNASeq provides for adjusting the LOESS and blacklisting parameters. Here the default settings were used. Copy number ratios were calculated, normalised to the median and outliers smoothed. Profiles were segmented using a Circular Binary Segmentation algorithm and aberrations called. The scale of aneuploidy as a percentage of the genome was calculated.
